# A non-linear analysis of Turing pattern formation

**DOI:** 10.1371/journal.pone.0220994

**Published:** 2019-08-09

**Authors:** Yanyan Chen, Javier Buceta

**Affiliations:** 1 Department of Bioengineering, Lehigh University, Iacocca Hall, Bethlehem, Pennsylvania, United States of America; 2 Department of Chemical and Biomolecular Engineering, Lehigh University, Iacocca Hall, Bethlehem, Pennsylvania, United States of America; Universita degli Studi di Firenze, ITALY

## Abstract

Reaction-diffusion schemes are widely used to model and interpret phenomena in various fields. In that context, phenomena driven by Turing instabilities are particularly relevant to describe patterning in a number of biological processes. While the conditions that determine the appearance of Turing patterns and their wavelength can be easily obtained by a linear stability analysis, the estimation of pattern amplitudes requires cumbersome calculations due to non-linear terms. Here we introduce an expansion method that makes possible to obtain analytical, approximated, solutions of the pattern amplitudes. We check and illustrate the reliability of this methodology with results obtained from numerical simulations.

## Introduction

Spatiotemporal pattern formation is an all-important and ubiquitous self-organization phenomenon in nature [1]. Processes in Physics, Chemistry, Biology, and even Social Sciences, display the formation of organized structures arising from the interaction of individual constituents [2–6]. In this regard, Alan Turing’s pioneering studies on pattern formation stands out among various mechanisms [7]. To begin with, it explains how one of the main transport mechanisms in nature with homogenizing properties, i.e. diffusion, can, counterintuitively, lead to the formation of inhomogeneous, ordered, structures. Notably, during the last decades different studies have revealed the widespread biological applicability of Turing’s ideas to different processes, such as animal coating [4, 8, 9], vertebrate limb development [10–12], tooth primordium patterning [4, 13], the ruggae spacing in the mammalian palate [14], the Min oscillations in bacteria [15, 16], or epidemiology and ecology problems [17–20].

The basic ingredients of a Turing instability are antagonistic local interactions between species with distinct diffusivity properties, e.g. activators vs. inhibitors in a chemical context or infected vs. susceptible individuals in epidemiology models,. Yet, not all combinations of the parameters result in a Turing instability (i.e. patterning). The regions in the parameter space leading to pattern formation, as well as the spatial periodicity, are usually determined by a linear stability analysis [4]. However, the estimation of other patterning properties, such as the amplitude or the pattern arrangement, require cumbersome calculations, e.g. the amplitude equations formalism [21, 22]. We point out that the pattern amplitude determines key features such as the maximum protein expression levels or the degree of infection during an epidemic. Consequently, having simplified means to estimate amplitude values and to understand how those are modulated by non-linearities is advantageous. Here we address this problem and introduce an expansion method that results in formulas to estimate the pattern amplitudes. Our analysis focuses, following Cross and Hohenberg’s pattern categorization [2], on the common *I*_*s*_ instabilities (periodic patterns in space that are stationary in time) and we illustrate our findings using the case of two interacting species.

## A non-linear analysis of the Turing instability

For the sake of simplicity, we restrict our analysis to two coupled reaction-diffusion equations in one spatial dimension. Thus, let us consider the following set of equations describing the spatiotemporal dynamics of two scalar fields *u* = *u* (*x*, *t*) and *v* = *v* (*x*, *t*),

$$\begin{array}{ccc} \frac{\partial u}{\partial t} & = & f(u,v)+\frac{\partial^{2}u}{\partial x^{2}}\\ \frac{\partial v}{\partial t} & = & g(u,v)+D_{v}\frac{\partial^{2}v}{\partial x^{2}} \end{array}$$ (1)

The functions *f* and *g* (reactive terms) are supposed to have an equilibrium point, $P_{0}=(u^{0},v^{0})$, that defines a steady, pattern-free (i.e. homogeneous) solution such that *f*(*u*^0^, *v*^0^) = *g*(*u*^0^, *v*^0^) = 0. By defining $f_{z}^{0}=\frac{\partial f}{\partial z}{|}_{P_{0}}$ and $g_{z}^{0}=\frac{\partial g}{\partial z}{|}_{P_{0}}$, where *z* stands for either the field *u* or *v*, the equilibrium point is stable if,

$$\begin{array}{ccc} f_{u}^{0}+g_{v}^{0} & < & 0\\ f_{u}^{0}g_{v}^{0}-f_{v}^{0}g_{u}^{0} & > & 0 \end{array}$$ (2)

If a *stationary* pattern (type *I*_*s*_) eventually develops we expect the field *z* (*x*) to have a stationary solution with a functional form,

$$\begin{array}{c} z(x)=z_{0}+\delta_{z}\;\text{cos}\;(qx) \end{array}$$ (3)

where $\delta_{z}\in R\cup ⊘$ stands for the amplitude of the pattern of the Fourier mode, *q*. By substituting this ansatz, Eq (3), into Eq (1) the system reduces to a set of ordinary differential equations,

$$\begin{array}{ccc} \frac{\partial u}{\partial t} & = & f(u,v)-q^{2}[u-u^{0}]\\ \frac{\partial v}{\partial t} & = & g(u,v)-D_{v}q^{2}[v-v^{0}] \end{array}$$ (4)

We notice that the solution $P=P_{0}$, i.e. the pattern-free solution, is always a *stationary* solution of Eq (4). Thus, the appearance of stationary patterns rely on the existence of solutions $P_{*}=(u^{*}\neq u^{0},v^{*}\neq v^{0})$ such that,

$$\begin{array}{ccc} f(u^{*},v^{*})-q^{2}[u^{*}-u^{0}] & = & 0\\ g(u^{*},v^{*})-D_{v}q^{2}[v^{*}-v^{0}] & = & 0 \end{array}$$ (5)

Alternatively, since $P_{*}=P_{0}+\delta$ with *δ* = (*δ*_*u*_, *δ*_*v*_), this condition can be written as a function of the pattern amplitudes,

$$\begin{array}{ccc} f(u^{0}+\delta_{u},v^{0}+\delta_{v})-q^{2}\delta_{u} & = & 0\\ g(u^{0}+\delta_{u},v^{0}+\delta_{v})-D_{v}q^{2}\delta_{v} & = & 0 \end{array}$$ (6)

with $\delta_{u},\delta_{v}\in R$.

Assuming that *f* and *g* are polynomial functions (or admit a polynomial expansion around $P_{0}$),

$$\begin{array}{ccc} f(u,v) & = & f_{u}^{0}(u-u_{0})+f_{v}^{0}(v-v_{0})+\epsilon_{f}{\left(u-u_{0}\right)}^{p}{\left(v-v_{0}\right)}^{r}\\ g(u,v) & = & g_{u}^{0}(u-u_{0})+g_{v}^{0}(v-v_{0})+\epsilon_{g}{\left(u-u_{0}\right)}^{s}{\left(v-v_{0}\right)}^{t} \end{array}$$ (7)

then Eq (6) read,

$$\begin{array}{ccc} (f_{u}^{0}-q^{2})\delta_{u}+f_{v}^{0}\delta_{v} & = & -\epsilon_{f}\delta_{u}^{p}\delta_{v}^{r}\\ g_{u}^{0}\delta_{u}+(g_{v}^{0}-D_{v}q^{2})\delta_{v} & = & -\epsilon_{g}\delta_{u}^{s}\delta_{v}^{t} \end{array}$$ (8)

where *p* + *r* > 1 and *s* + *t* > 1. By defining *τ* = *ϵ*_*g*_/*ϵ*_*f*_, if we further assume that one of the non-linearities is dominant (i.e., |*τ*| < 1), we can perform a perturbative expansion of the pattern amplitudes $\delta_{z}=\sum_{i=0}\delta_{z}^{\left(i\right)}\tau^{i}$ such that at orden *τ*^0^ Eq (8) read,

$$\begin{array}{c} (f_{u}^{0}-q^{2})\delta_{u}^{\left(0\right)}+f_{v}^{0}\delta_{v}^{\left(0\right)}=-\epsilon_{f}{\delta_{u}^{\left(0\right)}}^{p}{\delta_{v}^{\left(0\right)}}^{r} \end{array}$$ (9)

$$\begin{array}{ccc} g_{u}^{0}\delta_{u}^{\left(0\right)}+(g_{v}^{0}-D_{v}q^{2})\delta_{v}^{\left(0\right)} & = & 0 \end{array}$$ (10)

Eq (10) can be interpreted in terms of a phase between *u* and *v* such that if $(\frac{g_{v}^{0}-D_{v}q^{2}}{g_{u}^{0}})>0$ then *u* and *v* patterns develop in a counter-phase manner. Eqs (9) and (10) can be solved and we obtain the following solutions for $\delta_{u}^{\left(0\right)}$ and $\delta_{v}^{\left(0\right)}$,

$$\begin{array}{ccc} \delta_{u}^{\left(0\right)} & = & ({\left(-1\right)}^{1-r}{\left(\frac{g_{v}^{0}-D_{v}q^{2}}{g_{u}^{0}}\right)}^{r}\frac{(f_{u}^{0}-q^{2})(g_{v}^{0}-D_{v}q^{2})-f_{v}^{0}g_{u}^{0}}{\epsilon_{f}(g_{v}^{0}-D_{v}q^{2})}{)}^{\frac{1}{p+r-1}}\\ \delta_{v}^{\left(0\right)} & = & ({\left(-1\right)}^{p}{\left(\frac{g_{v}^{0}-D_{v}q^{2}}{g_{u}^{0}}\right)}^{1-p}\frac{(f_{u}^{0}-q^{2})(g_{v}^{0}-D_{v}q^{2})-f_{v}^{0}g_{u}^{0}}{\epsilon_{f}(g_{v}^{0}-D_{v}q^{2})}{)}^{\frac{1}{p+r-1}} \end{array}$$ (11)

At order *τ*^1^ Eq (8) read,

$$\begin{array}{ccc} (f_{u}^{0}-q^{2})\delta_{u}^{\left(1\right)}+f_{v}^{0}\delta_{v}^{\left(1\right)} & = & -\epsilon_{f}(p{\delta_{u}^{\left(0\right)}}^{p-1}{\delta_{v}^{\left(0\right)}}^{r}\delta_{u}^{\left(1\right)}+r{\delta_{u}^{\left(0\right)}}^{p}{\delta_{v}^{\left(0\right)}}^{r-1}\delta_{v}^{\left(1\right)})\\ g_{u}^{0}\delta_{u}^{\left(1\right)}+(g_{v}^{0}-D_{v}q^{2})\delta_{v}^{\left(1\right)} & = & -\epsilon_{f}{\delta_{u}^{\left(0\right)}}^{s}{\delta_{v}^{\left(0\right)}}^{t} \end{array}$$ (12)

that lead to the solution,

$$\begin{array}{ccc} \delta_{u}^{\left(1\right)} & = & \frac{\epsilon_{f}{\delta_{u}^{\left(0\right)}}^{s}{\delta_{v}^{\left(0\right)}}^{t}(f_{v}^{0}+\epsilon_{f}r{\delta_{u}^{\left(0\right)}}^{p}{\delta_{v}^{\left(0\right)}}^{r-1})}{\left(1-p-r)((f_{u}^{0}-q^{2})(g_{v}^{0}-D_{v}q^{2})-f_{v}^{0}g_{u}^{0}\right)}\\ \delta_{v}^{\left(1\right)} & = & -\frac{\epsilon_{f}{\delta_{u}^{\left(0\right)}}^{s}{\delta_{v}^{\left(0\right)}}^{t}(f_{u}^{0}-q^{2}+\epsilon_{f}p{\delta_{u}^{\left(0\right)}}^{p-1}{\delta_{v}^{\left(0\right)}}^{r})}{\left(1-p-r)((f_{u}^{0}-q^{2})(g_{v}^{0}-D_{v}q^{2})-f_{v}^{0}g_{u}^{0}\right)} \end{array}$$ (13)

Thus, up to order $O(\tau^{2})$ the solution for the pattern amplitudes is given by $\delta_{z}=\delta_{z}^{\left(0\right)}+\tau \delta_{z}^{\left(1\right)}$ with $\delta_{z}^{\left(0\right)}$ and $\delta_{z}^{\left(1\right)}$ as prescribed by Eqs (11) and (13) respectively.

In case a pattern develops we expect to do it continuously, that is, with an amplitude growing from zero. Thus, the above solution must contain the trivial solution *δ*_*u*_ = *δ*_*v*_ = 0 that defines the patterning separatrix in the parameter space. The latter implies that a pattern develops if there exist a value of *q* for which *δ*_*z*_ = 0, that in turn requires that $\delta_{z}^{\left(0\right)}=0$ and leads to the condition $P(q^{2})=(f_{u}^{0}-q^{2})(g_{v}^{0}-D_{v}q^{2})-f_{v}^{0}g_{u}^{0}=0$. We stress that regardless of *P*(*q*^2^) appearing in the denominator of the expression for $\delta_{z}^{\left(1\right)}$ (see Eq (13)), one can easily check that no singularity develops due to the dependence of the numerator on $\delta_{z}^{\left(0\right)}∼{\left(P(q^{2})\right)}^{\frac{1}{p+r-1}}$. We also point out that lim_*q*^2^→∞_ *P*(*q*^2^) → +∞ and, by construction, *P*(0) > 0 (see Eq (2)). Thus, since *P*(*q*^2^) has an absolute minimum at ${q^{*}}^{2}=1/2(f_{u}^{0}+g_{v}^{0}/D_{v})$, the condition of existence of a patterning separatrix can be stated as *P*(q*^2^) = 0, that is,

$$\begin{array}{c} D_{v}f_{u}^{0}+g_{v}^{0}={\left[D_{v}(f_{u}^{0}g_{v}^{0}-f_{v}^{0}g_{u}^{0})\right]}^{1/2} \end{array}$$ (14)

That is, patterns develop if $D_{v}f_{u}^{0}+g_{v}^{0}\geq {\left[D_{v}(f_{u}^{0}g_{v}^{0}-f_{v}^{0}g_{u}^{0})\right]}^{1/2}$. Moreover, the most unstable Fourier mode of the developing pattern can be approximated by q* close to the separatrix. We point out that inside the patterning region a full band of Fourier modes becomes unstable and the fastest growing mode deviates from q*. More importantly, a coupling between Fourier components develops and such phenomenon is incompatible with the hypothesized functional form of the field (see Eq (3)). Consequently, we expect our results to be strictly valid in the vicinity of the patterning separatrix, and to obtain disagreements away from it. As for the role played by non-linear terms, Eq (14) is the same condition that is obtained using the conventional Fourier decomposition method over the linearized system (c.f. [4, 23]). Consequently, non-linear components are irrelevant for determining the boundaries of the patterning region as expected.

## Example: An activator-inhibitor model system

In order to test our analytical predictions, we consider the following example where *f* and *g*, as prescribed by Eq (7), read,

$$\begin{array}{ccc} f(u,v) & = & a(u-u^{0})+a(v-v^{0})-\frac{1}{2}{\left(u-u^{0}\right)}^{3}{\left(v-v^{0}\right)}^{2}\\ g(u,v) & = & -2(u-u^{0})-(v-v^{0})-\frac{1}{8}(u-u^{0})(v-v^{0}) \end{array}$$ (15)

with *a* > 0. This family of non-linear equations can be mapped into an activator-substrate model proposed to describe pigmentation patterns in sea shells [24] and into an activator-inhibitor model that accounts for the regeneration process in hydra [25]. Here we considered their linearized parts and included phenomenological non-linear terms with *p* = 3, *r* = 2, and *s* = *t* = 1 (see Discussion).

In this case, there is a single real equilibrium point of the reactive terms *f*(*u*^0^, *v*^0^) = *g*(*u*^0^, *v*^0^) = 0: $P_{0}=(u^{0},v^{0})$, that defines the steady, pattern-free solution and, since $\{g_{u}^{0},g_{v}^{0}\}<0$, $\{f_{u}^{0},f_{v}^{0}\}>0$, $f_{u}^{0}<\|g_{v}^{0}\|$, and $f_{u}^{0}\|g_{v}^{0}\|<f_{v}^{0}\|g_{u}^{0}\|$, then the stability conditions of $P_{0}$, as given by Eq (2), are automatically satisfied. Patterns develop if the following conditions hold (see Eq (14)),

$$\begin{array}{ccc} 1 & \geq & a\\ D_{v} & \geq & \frac{3+2\sqrt{2}}{a} \end{array}$$

Under those conditions, close the separatrix the periodicity of the pattern is given by the Fourier mode q*^2^ = (*a* − 1/*D*_*v*_) / 2 and the amplitudes by $\delta_{z}=\delta_{z}^{\left(0\right)}+\frac{1}{4}\delta_{z}^{\left(1\right)}$ with $\delta_{z}^{\left(0\right)}$ and $\delta_{z}^{\left(1\right)}$ as prescribed by Eqs (11) and (13) respectively. Thus, the leading order of the amplitudes reads,

$$\begin{array}{ccc} \delta_{u}^{\left(0\right)} & = & \frac{1}{2}{\left(\frac{\left(1+aD_{v})(1+aD_{v}(aD_{v}-6)\right)}{D_{v}}\right)}^{\frac{1}{4}}\\ \delta_{v}^{\left(0\right)} & = & 2{\left(\frac{1+aD_{v}(aD_{v}-6)}{D_{v}{\left(aD_{v}+1\right)}^{3}}\right)}^{\frac{1}{4}} \end{array}$$ (16)

In order to check our predictions, we ran numerical simulations using the Method of Lines (MOL) with periodic boundary conditions [26] implemented through a Wolfram’s Mathematica code [27] (S1 Simulation Code). For every value of the parameters *a* and *D*_*v*_ we changed the system size, *L*, such that 6 pattern wavelengths fitted into the spatial domain, i.e., $L=6\lambda =6\cdot 2\pi /{q^{*}}^{\frac{1}{2}}$. The initial conditions for *u* and *v* were periodic functions with a periodicity λ to facilitate reaching a stationary state faster.

**Fig 1:**
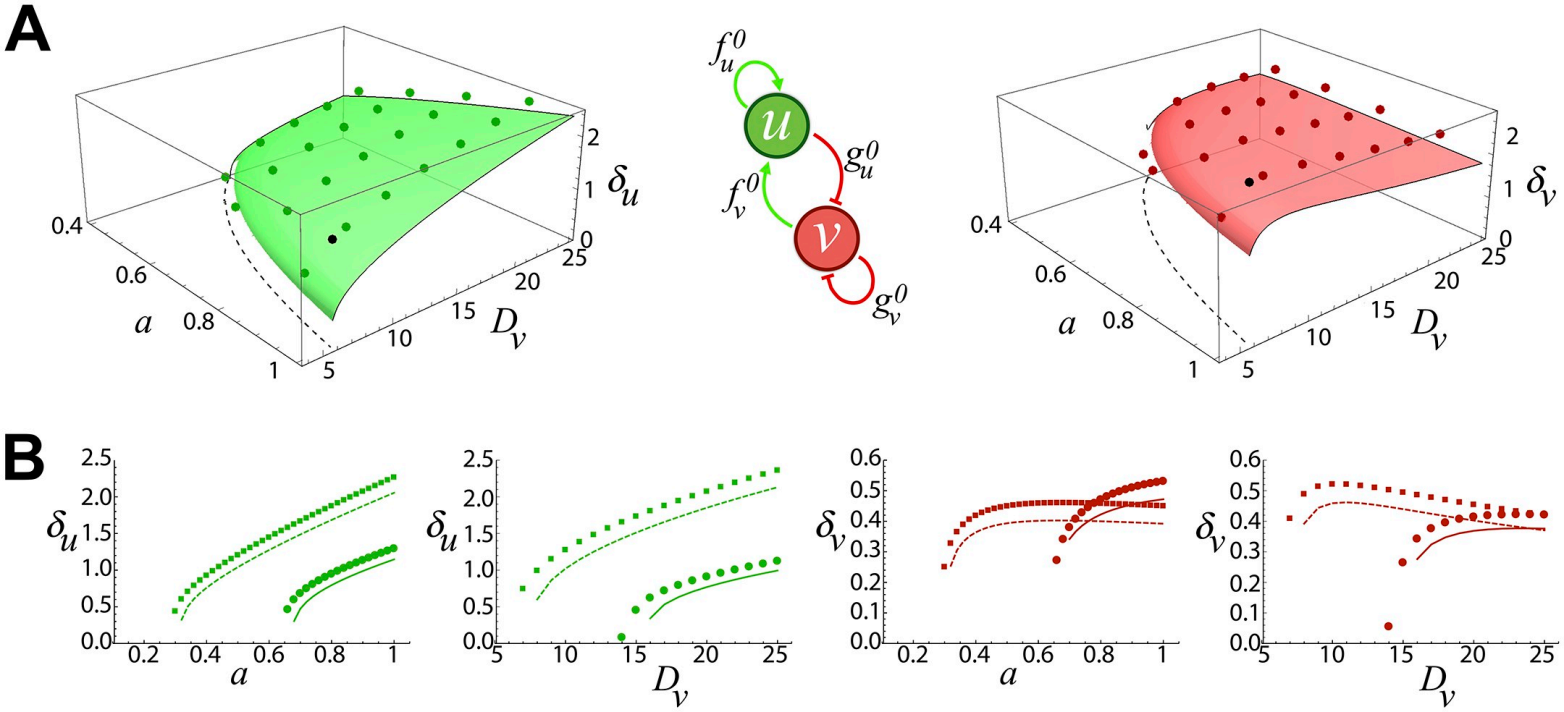
**A**: Amplitudes of the pattern *δ*_*u*_ (left) and *δ*_*v*_ (right) in the parameter space *a*-*D*_*v*_ obtained by our approach (continuous surfaces) and in numerical simulations (circles) for the activator, *u* (green in all panels), inhibitor, *v*, (red in all panels) system described in the main text (Eq 15). The black dashed line indicates the patterning separatrix, Eq (14), and the black circle indicates the parameter values used to obtain the pattern shown in Fig 2A. The central inset shows, graphically, the regulatory interactions between the species *u* and *v* as defined by the sign of the linear components, $f_{u}^{0}$, $f_{v}^{0}$, $g_{u}^{0}$, and $g_{v}^{0}$. **B**: Trajectories to sample the theoretical solutions. The symbols indicate simulations results and the lines the approximated solutions for fixed values of *D*_*v*_ = 9, 20 (solid and dashed lines respectively) and increasing *a*, and for fixed values of *a* = 0.4, 0.9 (solid and dashed lines respectively) and increasing values of *D*_*v*_.

**Fig 2:**
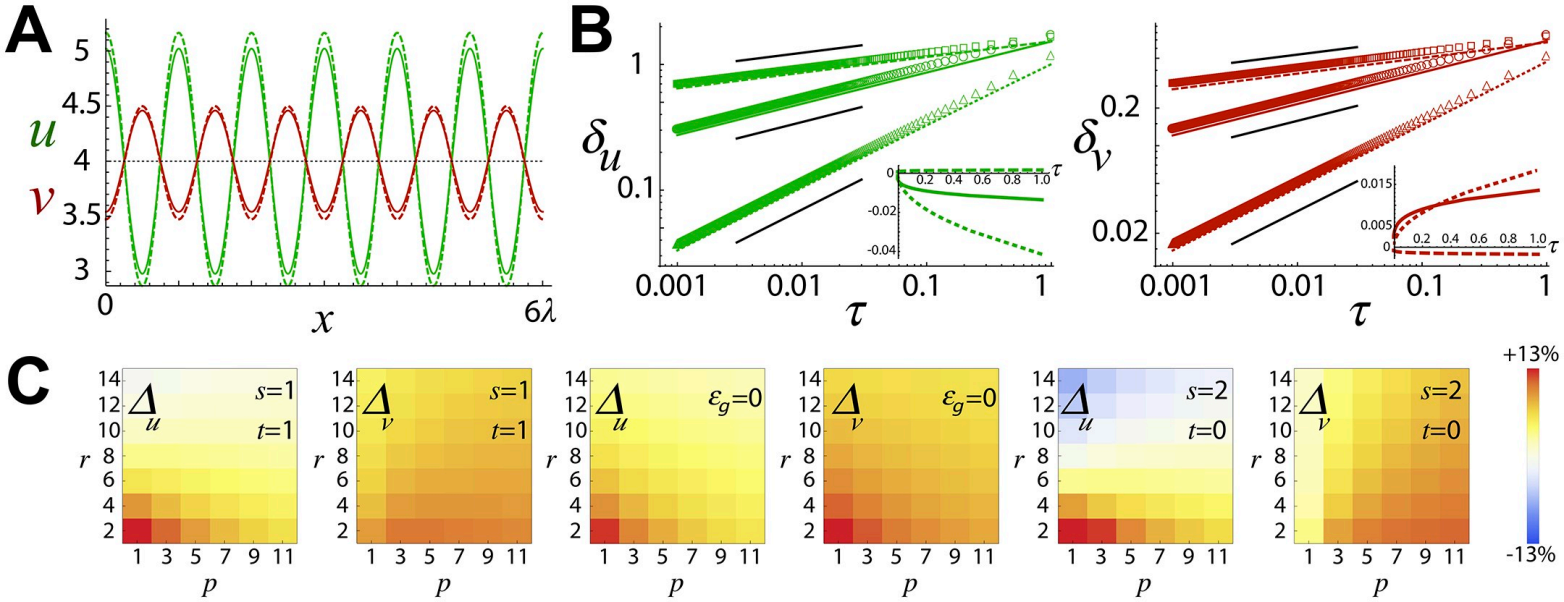
**A**: Stationary pattern solutions, *u* (green) and *v* (red), obtained by our theoretical approach (continuous lines) and in numerical simulations (dotted lines). Parameters: *a* = 0.9, *D*_*v*_ = 9 (black circle in Fig 1A). **B**: Pattern amplitudes as a function of the nonlinear parameter, *τ*, for different values of *p* and *r* (log-log axes): *p* = 3 and *r* = 2 (circles), *p* = 3 and *r* = 0 (triangles), and *p* = 5 and *r* = 4 (squares). The symbols stand for numerical results and the colored lines for the analytical solutions: *p* = 3 and *r* = 2 (solid line), *p* = 3 and *r* = 0 (dotted line), and *p* = 5 and *r* = 4 (dashed line). The black solids lines are a guide to the eye and show, from top to bottom, the functions $\tau^{\frac{1}{8}}$, $\tau^{\frac{1}{4}}$, and $\tau^{\frac{1}{2}}$. The insets show the relative importance of the first order correction versus the leading order of the expansion by plotting $\frac{\tau \delta_{z}^{\left(1\right)}}{\delta_{z}^{\left(0\right)}}$ as a function of *τ* (lines codes as in the main figure). **C**: Quantification of the relative error, Δ_*z*_, as a function of different combinations of the values of the exponents *p*, *r*, *s*, and *t* (the rest of parameters as in panel **A**). The scale bar (right) is the same for all density plots.

Fig 1 shows that the comparison between the approximated theoretical amplitudes, up to order $O(\tau^{2})$, and those obtained numerically in the parameter space *a* and *D*_*v*_. We point out that in Fig 1A the theoretical surface grows continuously from the patterning separatrix but such detail was skipped in the plot to perceive better the boundary line. We observed a fair quantitative agreement (see quantification in Fig 2) and the theoretical solutions captured both the behavior and the values of the pattern amplitudes as a function of *a* and *D*_*v*_. We noticed that for this particular choice of equations and parameters the theoretical solutions always underestimated the exact (i.e. numerical) solution. Yet, we found that for some cases of the non-linear exponents the theoretical solution overestimated the exact solution (see Fig 2).

The pattern solutions obtained by the theoretical approximation for *q* = q*, Eq (3), are indeed in agreement with those obtained in numerical simulations, Fig 2A. We also checked the accuracy of the theoretical approach to capture the power-law functional dependence of the pattern amplitude with *ϵ*_*f*_: note that the theoretical solution predicts that the dominant term of the pattern amplitude behaves as, $\delta_{z}^{\left(0\right)}∼\epsilon_{f}^{\frac{1}{1-p-r}}$. To that end, we ran simulations keeping constant *ϵ*_*g*_ = 1/10 and varying *ϵ*_*f*_ using different values of *p* and *r*. In this way, by plotting the values of the amplitudes as a function of the expansion parameter, *τ* = *ϵ*_*g*_/*ϵ*_*f*_, we validated both the scaling behavior and the goodness of the theoretical prediction as *τ* increases (Fig 2B). The results revealed an excellent agreement and showed that, in these particular examples, the estimations were accurate even for values of *τ* close to one since the first order correction is very small with respect the leading order $\delta_{z}^{\left(0\right)}$.

Finally, we investigated the accuracy of the theoretical approach as a function of different combinations of the values of the exponents *p*, *r*, *s*, and *t* (Fig 2C). To that end, we quantified the relative error by means of $\Delta_{z}=100\times (\delta_{z}^{num.}-\delta_{z}^{theo.})/\delta_{z}^{num.}$. We found that the error was always below 15% (absolute value). While for the different studied cases was not possible to found an overall trend, we found monotonic behaviors as a function of *p* and *r* and data revealed that increasing *r* systematically decreased the error. Also, as mentioned above, in most cases the theoretical solutions underestimate the exact solution (red-ish colors). Yet, some combination of exponents led to an overestimation of the numerical amplitude (blue-ish colors). To evaluate the relevance of truncating the expansion series at order $O(\tau^{2})$, we repeated our calculations for the case *ϵ*_*g*_ = 0. We point out that in that case the leading order, $\delta_{z}^{\left(0\right)}$, are the exact solution of Eq (8). As shown in Fig 2B, we did not find a perfect agreement between the numerical solution and the theoretical estimate. In fact, the relative errors for some values of the non-linear exponents are slightly larger than those found when *ϵ*_*g*_ ≠ 0 (cf. columns Δ_*u*_, 1 and 3, and columns Δ_*v*_, 2 and 4). This phenomenon can be explained as follows: as mentioned above, the hypothesis about the functional form of the patterning solution, Eq (3), breaks down as soon as we move away from the patterning boundary since a band of Fourier modes, and not just one as we assumed, becomes unstable. In any case, as suggested by Fig 2B, additional terms in the *τ*-expansion are not expected to increase significantly the accuracy of the theoretical estimation when *ϵ*_*g*_ ≠ 0.

## Discussion and conclusions

Here we have introduced a framework to analyze *I*_*s*_ patterns driven by a Turing instability. This approach maps reaction-diffusion systems into algebraic problems and makes possible to estimate approximated patterning solutions. Importantly, our method simplifies the cumbersome calculations required to obtain information about the amplitude of patterns. As for the limitations of our approach, the method assumes that the amplitude that corresponds to the most unstable Fourier mode represents accurately the real amplitude of the pattern. As shown here, this assumption leads to quantitative disagreements that are expected to be relevant far away from the patterning boundary. However, by comparing the predicted patterning solution obtained by this methodology with numerical solutions, we have shown that the former reproduces qualitatively, and, to a great extent, also quantitatively, the actual patterning solutions and their functional dependence on the model parameters. As a matter of discussion, our approach accounts for a single non-linear term in each reaction such that one of them is dominant. While this is certainly a limitation, a number of systems fall within that category. Some of them are models about gene networks [28] where the experimental evidence suggests the leading, linear, interactions and non-linearities are phenomenologically included to induce the saturation of the growing pattern, e.g. digit patterning during limb development [10]. Other models are those arising from enzyme kinetics studies where Hill-like regulatory functions are approximated by polynomial functions: e.g. the Selkov model of glycolysis [29, 30]. Finally, in some models, while the requirement about a single non-linearity in each reaction is fulfilled, the non-linear terms are equally dominant, i.e. *τ* = 1, for example the Schnakenberg model [31]. Still, given that our approach could still be applied under those conditions, see Fig 2B, we argue that even in those cases our methodology may hold. Possible extensions of the method, out of the scope of this manuscript, may include generalizations to other patterning situations, such as non-stationary patterns, or the effect of stochastic perturbations. Altogether, we hope that the methodology introduced here can be useful to analyze Turing patterning systems in different fields.

## Supporting information

S1 Simulation Code Mathematica code to perform simulations of Turing patterns. (NB)
